# Impact of weight loss on sexual and psychological functions and quality of life in females with sexual dysfunction: A forgotten avenue

**DOI:** 10.3389/fpsyg.2023.1090256

**Published:** 2023-02-01

**Authors:** Gehan A. Abdelsamea, Mostafa Amr, Ahmed M. N. Tolba, Haitham O. Elboraie, Amir Soliman, Badr Al-Amir Hassan, Faten Ali, Doaa A. Osman

**Affiliations:** ^1^Faculty of Physical Therapy, Department of Physical Therapy for Women’s Health, Cairo University, Giza, Egypt; ^2^Faculty of Physical Therapy, Department of Physical Therapy for Women’s Health, Delta University for Science and Technology, Gamasa, Egypt; ^3^Faculty of Medicine, Department of Psychiatry, Mansoura University, Mansoura, Egypt; ^4^Faculty of Physical Therapy, Department of Basic Science, Delta University for Science and Technology, Gamasa, Egypt; ^5^Faculty of Medicine, Department of Psychiatry, Helwan University, Helwan, Egypt; ^6^Faculty of Medicine, Department of Public Health and Community, Delta University for Science and Technology, Gamasa, Egypt; ^7^Faculty of Physical Therapy, Department of Physical Therapy for Internal Medicine and Geriatrics, Delta University for Science and Technology, Gamasa, Egypt

**Keywords:** weight loss, obesity, female sexual dysfunction, psychological function, quality of life

## Abstract

**Objectives:**

This study aimed to evaluate the impact of weight loss on sexual and psychological health as well as quality of life in females with sexual dysfunction.

**Materials and methods:**

The study was done at Delta University for Science and Technology in Gamasa, Egypt, on 40 obese married females having sexual dysfunction. Their age ranged from 20 to 40 years old, with a mean of 28.98 ± 4.96 years. They followed a weight loss program in the form of diet regimen and physical training for 6 months. Anthropometric measures, Arabic Female Sexual Function Index (FSFI), Arabic version of Hospital Anxiety and Depression Scale (HADS), and Arabic version of Short-Form 36 Health Survey (SF-36) were evaluated prior to starting the study, after 3 and 6 months of the study.

**Results:**

Statistical analysis revealed significant reductions in anthropometric measures, as well as significant improvements in HADS and SF-36 scores after both 3 and 6 months of weight loss intervention compared to the baseline measurements, while there were significant improvements in sexual arousal, lubrication, patient satisfaction as well as the total score of FSFI after 3 months and contrarily there were no statistically significant changes in any of the FSFI’s domains or overall score after 6 months of the weight loss program compared to baseline.

**Conclusion:**

Weight loss improves females’ anthropometric measures, psychological function and quality of life; however, it has no direct effect on female sexual dysfunction (FSD) after 6 months compared to baseline, so increased awareness of FSD is necessary as this issue suffers from inadequate identification and management.

## Introduction

The prevalence of obesity has multiplied several folds in developing nations, including Egypt. Regarding the World Health Organization’s estimate of worldwide illness prevalence, Egypt is rated 18th with the greatest prevalence of obesity (ProCon.org, 2020). Regarding the 2019, 100 million health survey, the prevalence of obesity in adults in Egypt has climbed to around 40% from the predicted 36% in the 2017 STEPwise report. Egyptian women are more likely than men to be obese (about 50% compared to 30%, respectively) (Aboulghate et al., 2021).

Estimating the burden of obesity is especially challenging since it is not a direct impact of obesity but rather a result of several co-morbidities. There has been very few research published about the burden of diseases in Egypt (Abolfotouh et al., 2008). Obesity is accompanied with numerous comorbidities, including diabetes mellitus, hypertension, cardiovascular diseases, joint and motion problems, pulmonary problems including asthma, and mental health problems like anxiety and depression (Pi-Sunyer, 2009; Fruh, 2017).

Obesity’s influence on sexual function has received little attention. However, the limited research that is available suggests that obesity appears to have a negative impact on sexual functioning (Kolotkin et al., 2006; Mozafari et al., 2015; Shah et al., 2021). Obesity causes an imbalance of sexual hormone levels, leading to reduced sexual desire, arousal, and orgasm (Shah et al., 2021). Additionally, it increases a person’s chance of developing cardiometabolic conditions like type 2 diabetes, dyslipidemia, and hypertension, which are all associated with decreased sexual activity (Lyall et al., 2017). Moreover, obese individuals are more prone to experience anxiety and/or depression issues, which are thought to be related to sexual function directly or indirectly (Mather et al., 2009).

In the past, sexual dysfunction was thought to be a condition that exclusively affected men, but today it is recognized as a serious health issue for women as well (Aslan and Fynes, 2008). Female sexual dysfunction (FSD) is described as a disorder of desire, arousal, orgasm, pain, lubrication, and satisfaction in females (Kingsberg and Althof, 2009). The prevalence of sexual dysfunction in all women ranges from 25 to 63% (Jaafarpour et al., 2013). Obese women have a prevalence of FSD that varies from 50 to 86% (Yaylali et al., 2010), as the higher body mass index (BMI) is accompanied by greater impairment of sexual quality of life in women (Kolotkin et al., 2006).

Although sexual function promotion in obese women having sexual dysfunction represents a very important topic in women’s health care (Karimi et al., 2020), there is very little evidence on the influence of weight loss only on sexual function improvement, particularly in the Arab world. Therefore, this study aimed to evaluate the effect of a weight loss program on sexual function, psychological health, and quality of life among a sample of obese Egyptian married women having sexual dysfunction.

### Research hypothesis

It was hypothesized that there was no impact of weight loss on sexual and psychological functions and quality of life in females with sexual dysfunction.

## Materials and methods

### Design

The study was designed as a within-subject design. Before the study began, the Faculty of Physical Therapy, Cairo University’s Institutional Review Board approved the research conduction (No: P.T.REC/012/003569). The Helsinki Declaration Principles for Human Research were taken into account in this study. The participants provided their written informed consent to participate in this study. It was conducted from January to August 2022.

### Participants

A sample of 40 obese married females was recruited through flyers and social media invitations from the obesity clinic of the diet center of the faculty of physical therapy at Delta University for Science and Technology, Dakahlia, Gamasa, Egypt, after confirming their diagnosis of FSD by the Arabic Female Sexual Function Index (FSFI). Each participant signed a written informed consent guaranteeing confidentiality after being informed about the study’s purpose and methodology as well as the right they have to withdraw their consent at any moment throughout the study.

### Eligibility criteria

All participants were selected as obese, non-smoking, married women having sexual dysfunction (FSFI’s score < 28.1) and seeking weight loss programs for cosmetic purposes only. Their ages ranged from 20 to 40 years with a BMI of 30 kg/m^2^ or above, and a parity number ranged from 1 to 3 times. The exclusion criteria were polycystic ovarian disease, any gynecological disease, fibroids, pelvic surgery or radiotherapy, anemia, diabetes, hypertension, cardiovascular disease, thyroid disease, neurological disorder, rheumatoid arthritis, bariatric surgery, or taking weight loss medication. Also, women who had not engaged in sexual activity in the previous 6 months, as well as those who were pregnant or breastfeeding, were excluded from the study.

### Interventions

All women received a weight loss program in the form of a diet regimen plus physical training for 6 months.

#### Diet regimen

All participants followed a diet regimen created by the same nutrition-certified physical therapist. In the beginning, the Harris–Benedict equation was multiplied by 1.4 to calculate the total energy requirement. The daily caloric intake was subsequently reduced by 500 kcal per day (daily caloric intake = daily total energy requirement–500 kcal) (Thompson and Noel, 2016). A balanced diet of 55% carbohydrates, 30% fat, as well as 15% proteins was maintained by eating several modest meals every day. The recommended minimum daily fluid intake was set at 2,000 ml. Follow up visits were conducted every week to monitor how well they adhered to the nutritional rehabilitation program, calculate the new caloric requirement of each participant, and modify the diet chart selectively for each case.

#### Physical training

All participants exercised according to an escalating schedule, 3 days/week, for 6 months. They exercised, after eating breakfast by 2 h, at the obesity clinic of the diet center at faculty of physical therapy, Delta University for Science and Technology. Exercises included cycling on an ergometer for 10 min at 50–60 rpm, and walking on a treadmill for 20 min at an inclination of 0–3%.

### Outcome measures

#### Anthropometric measures

At the start of the program, at 3 and 6 months, every patient’s weight and height were measured using a weight-height scale. Participants were asked to remove some layers of clothing and go barefoot for the measurement process. The BMI was then determined by dividing the subject’s weight by their squared height.

#### Sexual function assessment

The Arabic FSFI was utilized to confirm the diagnosis of FSD before entering into the study and to assess the sexual function at baseline and after 3 and 6 months of the weight loss program. The FSFI is a 19-item self-report instrument that is both brief and multidimensional in its ability to assess essential aspects of women’s sexual function. Desire, arousal, lubrication, orgasm, patient satisfaction, as well as pain are the six dimensions of sexual function evaluated. The FSFI questionnaire has five possible answers for the first two questions, then zero to five for the remaining questions. The obtained scores are put together and multiplied by the appropriate factor (coefficients for questions 1, 2: 0.6, 3–10: 0.3, 11–19: 0.4). The range of the scoring system is from 2 to 36. A score above 28.1 suggested a healthy sexual life, whereas a score below 28.1 showed impaired sexual function (Anis et al., 2011). Sahar’s grading system was used to further categorize FSD severity into the five categories of severe (2–7.2), moderate (7.3–14.4), mild to moderate (14.5–21.6), mild (21.7–28.1 “cutoff point”), and no FSD (28.2–36) (Ismail et al., 2021).

The Arabic FSFI is a valid, reliable, and locally accepted instrument for assessing FSD in the population of Egypt. High test-retest reliability could be seen in the overall score and the scores of different domains (ranging from 0.92 to 0.98). High internal consistency was evident in the domains (ranging from 0.85 to 0.94). It was found that the Arabic FSFI had strong discriminant validity. The Arabic FSFI revealed an excellent overall performance [area under the curve (AUC) 0.985, 95% confidence interval (CI) 0.978–0.992] (Anis et al., 2011).

#### Psychological function assessment

The psychological function of each participant was evaluated by the Arabic version of Hospital Anxiety and Depression Scale (HADS) at baseline and after 3 and 6 months of the weight loss program. There are 14 items on this self-assessment scale, which includes both depression as well as anxiety dimensions: 7 items for each dimension, with a cutoff of eight for anxiety and nine for depression. Since the scores for each item ranged from 0 to 3, the person could receive a result for either anxiety or depression between zero to 21 (Terkawi et al., 2017).

The Arabic version of HADS is a reliable and valid tool to assess the mood states. Cronbach’s αs were 0.83 (95% CI: 0.79–0.88) for the HADS anxiety subscale and were 0.77 (95% CI: 0.7–0.83) for the HADS depression subscale. The majority of patients thought that the questions of the HADS were clear, easily understood, and covered all their concerns about their hospital anxiety and depression (Terkawi et al., 2017).

#### Quality of life assessment

The quality of life was evaluated for each participant by the Arabic version of the Short-Form 36 Health Survey (SF-36) at baseline and after 3 and 6 months of the weight loss program. The scale is intended to assess disease burden and to assess the patient’s quality of life as an indication of the patient’s health status. It consists of 36 questions that evaluate the following elements: physical functioning (the ability to care for oneself and perform daily tasks); role limitations caused by physical health problems (the impact of one’s physical health on one’s capacity to perform daily tasks); bodily pain (the level of pain experienced whilst performing daily tasks); general health perceptions (how one sees one’s own health); vitality (the capacity to carry out daily tasks); and social functioning. Scores ranged from zero (most affected) to one hundred (not affected) (Ware et al., 1993).

Most of the studies examining the SF-36 reliability have exceeded 0.80. Reliability estimates in the physical and mental areas are typically above 0.90 (Ware and Sherbourne, 1992; Ware et al., 1993; McHorney et al., 1994).

## Statistical analysis

SPSS software for Windows, version 26.0, was used to process the collected data (Chicago, IL, United States). The Shapiro–Wilk test was utilized to ensure that the data was distributed normally. A one-way repeated measures analysis of variance (ANOVA) with the time within-subject factor was used to evaluate any differences in the mean changes scores of every assessment time for the Arabic FSFI (Desire, arousal, lubrication, orgasm, patient satisfaction, pain, as well as overall score), the Arabic version of HADS (anxiety score and depression score), as well as the Arabic version of the SF-36 scale (Physical functioning, role functioning/physical, role functioning/emotional, energy/fatigue, emotional wellbeing, social functioning, pain, general health, and total score). For subsequent multiple comparisons, the *F* value was calculated utilizing Wilks’ lambda and *post-hoc* testing utilizing the Bonferroni correction. The significance level was established at *p* < 0.05 for all statistical tests.

## Results

### Participants’ baseline characteristics

**FIGURE 1:**
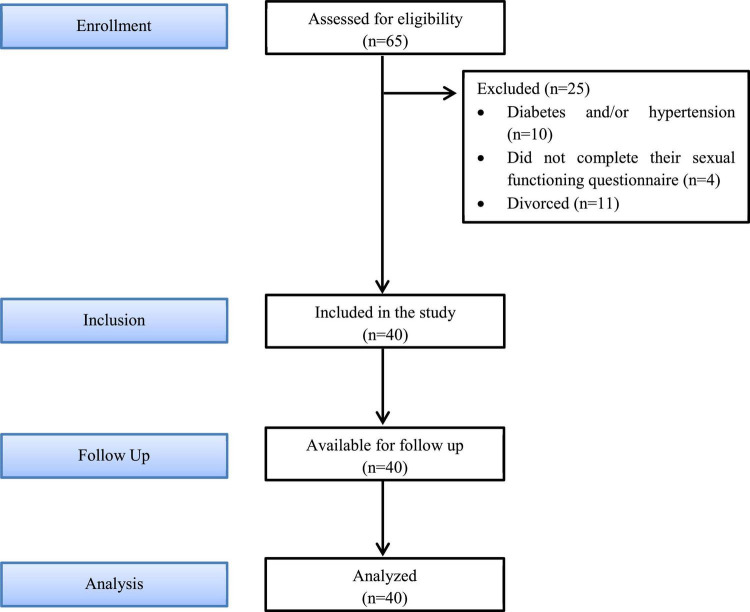
Flow-chart of participants.

A total of 65 females were investigated to be enrolled in this study. Baseline descriptive characteristics of the 40 women who met the criteria for this investigation are included in Table 1, while 25 participants were excluded from the study; 10 females had diabetes and/or hypertension, 4 females did not complete their sexual functioning questionnaire, and 11 females were divorced (Figure 1).

**TABLE 1:** Participants’ baseline characteristics.

Variable	Mean ± SD	Minimum	Maximum
Age (years)	28.98 ± 4.96	20.0	40.0
Height (m)	1.62 ± 0.05	1.55	1.72
Parity (score)	1.92 ± 0.65	1.0	3.0

SD, standard deviation; m, meter.

### Anthropometric measures

Repeated measure ANOVA revealed that the designed program had a significant effect on weight and BMI. For multiple comparisons between time intervals, the Bonferroni correction was used and revealed significant reductions in both weight and BMI at the three assessment times (Table 2).

**TABLE 2:** Anthropometric measures among the three assessment times.

	Baseline (Mean ± SD)	After 3 months (Mean ± SD)	After 6 months (Mean ± SD)	*p*-value
Weight (Kg)	87.90 ± 6.28	72.55 ± 5.43	68.90 ± 4.41	0.0001*
BMI (kg/m^2^)	33.19 ± 0.75	27.40 ± 1.00	26.03 ± 0.69	0.0001*
***Multiple pairwise comparisons (Bonferroni correction) for the anthropometric measures among the three assessment times***				
		**Baseline vs. after 3 months**	**Baseline vs. after 6 months**	**After 3 months vs. after 6 months**
Weight (Kg)		0.0001*	0.0001*	0.0001*
BMI (kg/m^2^)		0.0001*	0.0001*	0.0001*

BMI, body mass index; SD, standard deviation; p-value, probability; *significant with *p* < 0.05.

### Sexual function assessed by the Arabic FSFI

Repeated measure ANOVA found a significant effect of weight loss on arousal, lubrication, patient satisfaction domains, and overall score of FSFI. The Bonferroni correction which used for subsequent multiple comparisons between time intervals revealed that there were significant increases in arousal, lubrication, patient satisfaction domains, and overall score of FSFI after 3 months compared with baseline, while there were significant reductions in arousal, lubrication, patient satisfaction domains, and total score of FSFI after 6 months compared with after 3 months of intervention. The arousal, lubrication, patient satisfaction domains, and overall score of FSFI showed non-significant differences after 6 months compared with baseline (Table 3).

**TABLE 3:** Sexual function assessed by the Arabic FSFI among the three assessment times.

	Baseline (Mean ± SD)	After 3 months (Mean ± SD)	After 6 months (Mean ± SD)	*p*-value
Desire	1.84 ± 0.45	1.89 ± 0.42	1.92 ± 0.41	0.109
Arousal	1.57 ± 0.23	3.13 ± 0.35	1.62 ± 0.24	0.0001*
Lubrication	1.86 ± 0.38	3.66 ± 0.26	1.89 ± 0.32	0.0001*
Orgasm	1.50 ± 0.34	1.57 ± 0.51	1.60 ± 0.58	0.103
Satisfaction	1.47 ± 0.35	2.87 ± 0.35	1.51 ± 0.33	0.0001*
Pain	1.52 ± 0.46	1.53 ± 0.43	1.57 ± 0.43	0.132
Total score	9.77 ± 1.17	10.19 ± 1.07	9.81 ± 1.17	0.0001*
***Multiple pairwise comparisons (Bonferroni correction) for the significant domains of the Arabic FSFI among the three assessment times***				
		**Baseline vs. after 3 months**	**Baseline vs. after 6 months**	**After 3 months vs. after 6 months**
Arousal		0.0001*	0.082	0.0001*
Lubrication		0.0001*	0.07	0.0001*
Satisfaction		0.0001*	0.164	0.0001*
Total score		0.0001*	0.273	0.0001*

FSFI, Female Sexual Function Index; SD, standard deviation; p-value, probability; *significant with *p* < 0.05.

### Psychological function assessed by the Arabic version of HADS

Repeated measure ANOVA found a significant effect of weight loss on both anxiety and depression domains of the Arabic version of HADS. The Bonferroni correction revealed significant reductions in both anxiety and depression domains of the Arabic version of HADS at the three assessment times (Table 4).

**TABLE 4:** Psychological function assessed by the Arabic version of HADS among the three assessment times.

	Baseline (Mean ± SD)	After 3 months (Mean ± SD)	After 6 months (Mean ± SD)	*p*-value
Anxiety	8.40 ± 1.54	8.20 ± 1.30	7.47 ± 0.81	0.001*
Depression	18.47 ± 1.34	11.75 ± 1.23	6.15 ± 1.12	0.0001*
***Multiple pairwise comparisons (Bonferroni correction) for the Arabic version of HADS among the three assessment times***				
		**Baseline vs. after 3 months**	**Baseline vs. after 6 months**	**After 3 months vs. after 6 months**
Anxiety		0.01*	0.0001*	0.0001*
Depression		0.0001*	0.004*	0.0001*

HADS, Hospital Anxiety and Depression Scale; SD, standard deviation; p-value, probability; *significant with *p* < 0.05.

### Quality of life evaluated by the Arabic version of SF-36

Repeated measure ANOVA found a significant effect of weight loss on all domains of the SF-36 scale, except for the role functioning/physical domain that showed a non-significant effect. The Bonferroni correction revealed significant increases in all domains of the SF-36 scale at the three assessment times, except for the role functioning/physical domain that showed a non-significant differences at the three assessment times (Table 5).

**TABLE 5:** Quality of life assessed by the Arabic version of SF-36 among the three assessment times.

	Baseline (Mean ± SD)	After 3 months (Mean ± SD)	After 6 months (Mean ± SD)	*p*-value
Physical functioning	42.75 ± 4.52	47.37 ± 5.54	76.62 ± 5.70	0.0001*
Role functioning/Physical	100.0 ± 0.0	100.0 ± 0.0	100.0 ± 0.0	1.00
Role functioning/Emotional	0.0 ± 0.0	50.0 ± 50.63	78.33 ± 31.62	0.0001*
Energy/Fatigue	15.0 ± 14.14	40.0 ± 12.81	54.50 ± 12.80	0.0001*
Emotional wellbeing	18.0 ± 12.65	33.0 ± 12.44	47.0 ± 13.99	0.0001*
Social functioning	60.0 ± 12.40	71.87 ± 14.08	86.25 ± 12.60	0.0001*
Pain	95.50 ± 8.45	95.50 ± 8.45	98.50 ± 5.33	0.012*
General health	75.0 ± 0.0	77.12 ± 2.50	80.0 ± 0.0	0.0001*
Total score	406.25 ± 25.34	514.87 ± 60.52	621.21 ± 44.13	0.0001*
***Multiple pairwise comparisons (Bonferroni correction) for the Arabic version of SF-36 among the three assessment times***				
		**Baseline vs. after 3 months**	**Baseline vs. after 6 months**	**After 3 months vs. after 6 months**
Physical functioning		0.0001*	0.0001*	0.004*
Role functioning/Physical		1.00	1.00	1.00
Role functioning/Emotional		0.0001*	0.0001*	0.0001*
Energy/Fatigue		0.0001*	0.0001*	0.0001*
Emotional wellbeing		0.0001*	0.0001*	0.0001*
Social functioning		0.0001*	0.0001*	0.0001*
Pain		1.00	0.037*	0.037*
General health		0.0001*	0.0001*	0.019*
Total score		0.0001*	0.0001*	0.0001*

SF-36, Short-Form 36 Health Survey; SD, standard deviation; p-value, probability; *significant with *p* < 0.05.

## Discussion

The majority of overweight as well as obese women feel they lack the physical attractiveness, sexual interest, and emotional maturity to have a successful romantic relationship. As a result, they face a greater threat of sexual dysfunction than normal-weight women (Adolfsson et al., 2004).

In the current study, young and middle ages were specifically picked because they are associated with marriage and sexual activity, as well as their increased capacity to adhere to weight loss instructions, particularly exercise, and because these two age groups are more vulnerable to mental stress as a result of weight criticism (Edwards, 1994; Shalaby et al., 2020).

In the present study, weight loss after 3 months was accompanied with a substantial improvement in female sexual function, the improvement included considerable enhancement in three domains; sexual arousal, lubrication, and sexual satisfaction as well as the overall score, which is in accordance with inferences of similar previous studies which stated that weight reduction improves the females’ sexual arousal, lubrication, and satisfaction (Kolotkin et al., 2012; Aversa et al., 2013; Syed et al., 2021).

The improved sexual function after 3 months of weight loss might be attributed to the beneficial effects of losing weight on reducing adiposity and anthropometric parameters, which might improve a woman’s mobility, make it easier for her husband to penetrate her vagina, and give the couple more options when looking for comfortable positions during sexual activity (Oliveira et al., 2019). Additionally, losing weight may improve endothelial function, genital blood flow, and insulin resistance, all of which are variables that could improve female sexual function (Syed et al., 2021).

However, the results of our weight loss program after 6 months were unexpected as there were no statistically significant changes in any of the FSDI’s domains or overall score when compared to the baseline, indicating that weight loss alone does not directly affect a woman’s sexual function. This might be due to lack of sexual rehabilitation which is highly deficient in this region of the world rather than weight loss programs alone as the lack of education and awareness about sexual health contributes to the stigma that surrounds talking about sexuality and limitation of professional help by the females. Additionally, after losing a lot of weight, the loose skin that remains in regions like the stomach, the thighs, and the arms may lower the sexual attraction (Sarwer et al., 2005). Moreover, some women attribute the loss of their desire to overfamiliarity with their husbands, as well as lack of novelty and excitement in their sexual relationship (Sims and Meana, 2010). Furthermore, the practice of circumcision and other social and economic issues, including economic stress and increased home obligations, have been blamed by many women for their reduced sexual desire (El-Defrawi et al., 2001).

Concerning anxiety and depression, the findings of the present study showed that weight loss resulted in significant improvement of HADS score after both 3 and 6 months of weight reduction program, with the peak of improvement at 6 months, and the improvement in depression scores was much better than anxiety score. These came in agreement with the studies of Bulik et al. (2002), Irandoost et al. (2015), Carson et al. (2017), and Shalaby et al. (2020).

The substantial correlation between obesity and anxiety might be explained by the fact that people who are overweight often have low self-esteem, a lack of social and cultural support, as well as body image dissatisfaction, all of which can lead to anxiety disorders such as panic attacks and social phobia (van Vuuren et al., 2019).

The obvious connection between depression and obesity might be explained by the load of physical complications of obesity (Mulugeta et al., 2018; Sarigiani et al., 2020), in addition to the negative body image, lack of competence, relationship difficulties, and internalization of weight bias that are often linked to obesity (Sarigiani et al., 2020). Moreover, a positive relation had been found between depression and emotional eating (Konttinen et al., 2019).

Finally, concerning quality of life, our interventions for weight loss showed a substantial improvement in SF-36 scores after 3 and 6 months. This was similar to the results of the studies of Kaukua et al. (2003), Bryan et al. (2006), Olszanecka-Glinianowicz et al. (2014), Audureau et al. (2016), and Shalaby et al. (2020).

It is possible that satisfaction with behavior modification through exercise and a healthy diet could explain improvements in the SF-36 scores. Improvements might also have resulted through social interactions during program participation, weight loss intervention support from health staff, and participation in community intervention (Ambak et al., 2018). Moreover, the improvements in females’ anthropometric measures, anxiety scores, and depression scores reported in the current study could explain the improvement in their SF-36 scores.

## Strengths and limitations

One of the current study’s strengths is that it is one of the few Arabian studies that examined the impact of weight loss not only on FSD but also on psychological health as well as quality of life. Moreover, valid questionnaires were used to diagnose FSD, as well as to assess sexual and psychological functions and quality of life.

There are also several limitations to the current study. The current study’s main limitation is the limited sample size. The study’s smaller sample size was a direct result of its being limited to a single institution. Factors in the individual’s social environment, such as the woman’s age, her husband’s age, the length of her marriage, intercourse frequency, and household income were not taken into account, which could impact sexual satisfaction. Cases with major depression may need the use of some antidepressant medications before using non-invasive techniques (Girardi et al., 2009). Finally, more longitudinal studies were clearly needed in order to obtain more accurate and predictable data.

## Conclusion

Weight reduction has no direct effect on FSD after 6 months compared to the baseline measurement; the relationship between weight and sexual function misses several details and still unclear. On the other hand, 6 months of weight loss produces significant improvements in females’ anthropometric measures, psychological function and quality of life.

## Data availability statement

The raw data supporting the conclusions of this article will be made available by the authors, without undue reservation.

## Ethics statement

The Faculty of Physical Therapy, Cairo University’s Institutional Review Board approved the research conduction. The patients/participants provided their written informed consent to participate in this study.

## Author contributions

All authors contributed to the concept and design of the study, collected the data, and performed the statistical analysis and data interpretation, collaborated in writing and critical revision of the study, and read and agreed to the published version of the manuscript.
